# A single-cell genome perspective on studying intracellular associations in unicellular eukaryotes

**DOI:** 10.1098/rstb.2019.0082

**Published:** 2019-10-07

**Authors:** Tomáš Tyml, Shailesh V. Date, Tanja Woyke

**Affiliations:** 1 U.S. Department of Energy, Joint Genome Institute, Walnut Creek, CA, USA; 2 Global Viral, San Francisco, CA, USA

**Keywords:** single-cell genomics, cultivation bias, unicellular eukaryotes, intracellular associations, endosymbiont, giant viruses

## Abstract

Single-cell genomics (SCG) methods provide a unique opportunity to analyse whole genome information at the resolution of an individual cell. While SCG has been extensively used to investigate bacterial and archaeal genomes, the technique has been rarely used to access the genetic makeup of uncultivated microbial eukaryotes. In this regard, the use of SCG can provide a wealth of information; not only do the methods allow exploration of the genome, they can also help elucidate the relationship between the cell and intracellular entities extant in nearly all eukaryotes. SCG enables the study of total eukaryotic cellular DNA, which in turn allows us to better understand the evolutionary history and diversity of life, and the physiological interactions that define complex organisms.

This article is part of a discussion meeting issue ‘Single cell ecology’.

## 1. Introduction

Associations of eukaryotes with archaea and bacteria are central to eukaryote evolution and were the driving force behind the emergence of the eukaryotic cell. An alphaproteobacterial symbiont that once settled within a pre-eukaryotic cell gave rise to mitochondria [1]. Likewise, other bioenergetic organelles, primary plastids of Archaeplastida, arose upon a symbiotic event where an archaeplastid ancestor engulfed a cyanobacterium [2]. During later stages of evolution, some major eukaryotic lineages independently acquired secondary or even tertiary plastids, for instance by taking up a cell already containing the primary or secondary plastid, respectively [3]. Over time, the morphologies and genomes of precursors of secondary and tertiary plastids have been reduced to different degrees. Unlike most of the phototrophic eukaryotic lineages, chromistan and chlorarachniophyte algae contain a nucleomorph, a residual nucleus originating from engulfed eukaryotes that contain the primary plastid within their secondary plastids [4,5]. Dinotoms (Kryptoperidiniaceae, a small group of Dinoflagellata) also retained the engulfed nucleus and preserved the symbiont's mitochondria within dinotom plastids [6].

While the evolutionary impact of semiautonomous organelles becomes easily apparent, they are not the only DNA containing entities that contribute to the genomic complexity of eukaryotic cells. Eukaryotes frequently host archaeal, bacterial and even eukaryotic endocytobionts (endosymbionts living intracellularly). Note that we use symbiosis here in its broadest sense—that is, any intimate and constant interaction along the continuum between mutualism and pathogenicity and ranging from harmful to beneficial and from facultative to obligate [7]. Whereas some of these associations resemble the fate of the semiautonomous organelles, i.e. being present in whole host population and transmitted only vertically (e.g., *Perkinsela* in *Neoparamoeba*, [8]), other endocytobionts (and intracellular entities) are dependent on horizontal transmission and thus their presence and prevalence in host populations is difficult to predict and fluctuates, respectively. In phagocytosis capable eukaryotes, another intracellular, nucleic acid containing component, albeit transient, is represented by ingested prey items. Lastly, viruses as common obligate intracellular parasites are extremely diverse and ubiquitous; despite their generally rather small particle and genome sizes they play a major role in controlling their host populations, thereby impacting biogeochemical cycles across ecosystems [9]. Giant viruses, members of nucleocytoplasmic large DNA viruses, are comparable in size and complexity to other intracellular entities. They infect a wide range of eukaryotes, especially those capable of phagocytosis [10] and can host other viruses [11], and they are predicted to having evolved from smaller viruses through successive horizontal gene transfer of genetic material from their hosts [12].

All the above-mentioned intracellular entities contain nucleic acids, and thus, the pool of total cellular DNA in a single eukaryotic cell reflects this sum. Aside from general biological importance, this has practical implications when using single-cell genomics (SCG). Here we do not reflect on these cellular conglomerates as something that weakens the SCG data but as an exciting opportunity to study underexplored multipartite associations within eukaryotic cells. Here, we focus on unicellular eukaryotes and discuss compositions of various possible genomic pools within their single cells. We illustrate how the cultivation skew is limiting our current understanding of eukaryote intracellular associations and how emerging SCG provides a promising tool to enhance our knowledge of the genetic make-up of these associations.

## 2. Genome-wide approaches to studying intracellular associations in unicellular eukaryotes

**Figure 1.**
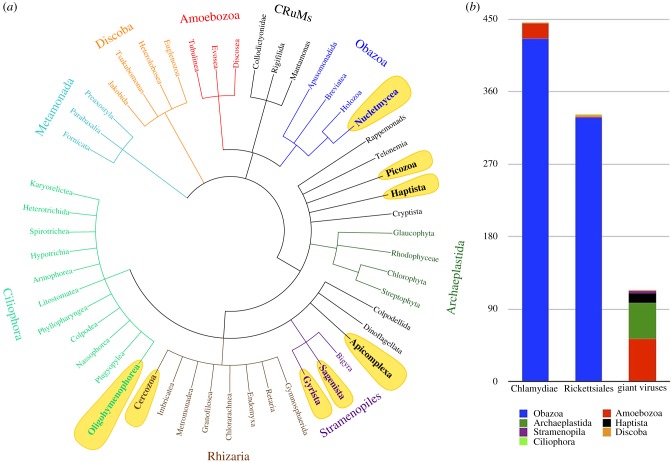
(*a*) Schematic relationships among the major eukaryotic groups (based on Adl *et al*. [21]). Lineages with intracellular entities identified by the SCG approach are in bold and highlighted in yellow. (*b*) Numbers of sequenced genomes of three groups of obligately intracellular parasites of eukaryotes (Chlamydiae, Rickettsiales, and giant viruses (defined here as members of double stranded DNA viruses with a genome greater than 0.2 Mb)) are displayed if their host is known (host clades are indicated by colour). Genomic data (genome size, host) were retrieved from JGI genome (http://genome.jgi.doe.gov) and NCBI genome (http://www.ncbi.nlm.nih.gov/genome) portals on 21 January 2019 or, when missing, from their respective publications.

Over the last decades, the use of easily-maintainable host laboratory cultures has been a standard procedure for studying eukaryote intracellular associations [13,14] and our understanding of the nature of many intracellular associations stems primarily from whole genome information. The need for genomic DNA of sufficient quantity and quality for studying interactions has narrowed the phylogenetic scope of research favouring model hosts grown axenically (i.e. isolated as single species, under contaminant free conditions) [15,16]. These laboratory model systems lack the higher complexity observed in naturally occurring intracellular associations; for example, associations between intracellular entities and a single host cell can vary across natural host populations [17]. Moreover, the genetic diversity of the intracellularly associated entities is expected to greatly exceed what can be deduced from laboratory culture-based models (e.g. [18,19]). Consequently, the effects of eukaryotic intracellular associations on ecosystem processes (through modulating microbial population structure [9]) or on their hosts (by providing new metabolic capabilities [20]) remain poorly studied beyond laboratory conditions. Looking at the phylogenetic diversity of eukaryotes that host three important obligate parasitic groups (Chlamydiae, Rickettsiales, giant viruses) studied at the genomic level (figure 1*b*), it is clear that Obazoa (which includes animals), Amoebozoa (namely *Acanthamoeba* strains), and Archaeplastida are overrepresented, suggesting that most of the diversity is still hidden and remains to be discovered. The biases and limitations of laboratory model systems can be partly overcome by employing cultivation-independent methods (e.g. metagenomics) on environmental samples; however, these methods lack single-cell resolution and therefore obscure host–symbiont relationships. Thus, only by focusing on one eukaryotic cell at a time can we obtain a detailed understanding of these fascinating multipartite associations and SCG has already begun to fill this gap (lineages labelled on figure 1*a*).

SCG has become a well-established approach for studying the coding potential and evolutionary histories of bacteria and archaea [22,23], specifically for microbial dark matter clades [24,25]. While bacteria and archaea have been extensively investigated by SCG, studies of associations within unicellular eukaryotes have lagged behind [26]. Unicellular eukaryotes are ubiquitous, encompass the most diverse and abundant part of the eukaryotic domain, and play a critical role across various ecosystems as primary producers, consumers, decomposers, and trophic links in food webs (e.g. [27]). Further, while some groups have initially used SCG (and SCG-like methods) to investigate this set of organisms, nearly all have followed protocols developed for the study of archaeal and bacterial associations (e.g. [28–32]; for the review of methods, see [23]). Although the standard SCG approach (i.e. physical separation of single cells and whole genome amplification and sequencing) works well with eukaryotes there are some unique aspects associated with sorting eukaryotic cells. Several separation techniques have been developed for obtaining individual cells (reviewed by Blainey [33]). Briefly, the separation techniques can be divided into two approaches based on whether the individual cells are selected randomly (random encapsulation methods) or identified first and then sorted (micromanipulation methods). The choice of the separation technique is of decisive influence on cell throughput and the micromanipulation approaches (e.g. micropipetting, optical tweezers) are among those considerably limited by their speed. Currently, the most common method, fluorescence-activated cell sorting (FACS), represents the random encapsulation approach and excels in speed. Another major advantage of FACS is the possibility of characterizing cells based on different fluorescence signals, e.g. autofluorescence from chlorophyll and specific staining for acidic organelles [28], providing some level of selectivity. The sizes of protists often make it possible to observe individual cells and their morphology under light microscopy, which enables: (i) assigning them to a group of target organisms [31]; (ii) examination of their current condition in fresh samples; or (iii) identifying the presence of endosymbionts and even their physical separation from the host cell [34]. On the technical side, sample preparation may have to be adjusted for some eukaryotic taxa compared to bacterial/archaeal samples. Generally, eukaryotic cells are much more fragile and sensitive to mechanical treatment (such as filtration or sonication) or cryopreservation, though this varies greatly across eukaryotic groups, e.g. Not *et al*. [35]. Thus, sample preparation needs to be tailored to meet the specific needs of a particular eukaryotic target group.

## 3. Single-cell genomics-enabled biological insights into eukaryote intracellular associations

The SCG approach has sufficient sensitivity to recover genomic data of other DNA containing entities present within a single cell. As referenced earlier, Yoon and colleagues [26] focused on an (at that time) obscure group of likely photoautotrophic marine plankton—Picobiliphyta (currently called Picozoa). Their data did not provide any evidence of plastid sequences, as was expected, but instead revealed bacterial and viral DNA probably originating from ingested prey items. Similarly, phagotrophic interactions were assumed in subsequent SCG-based studies (table 1). Plotnikov *et al*. [37] found rich bacterial assemblages within single cells of the ciliate *Paramecium aurelia*, however, it was not possible to unambiguously identify their role inside their hosts. Aside from the ingested prey, infections caused by nanovirus [26], and putative symbionts related to Rickettsiales and the candidate divisions ZB3 and TG2 [28] were also revealed. These and other studies provide an interesting perspective on the power of SCG and its limitations. SCG and ancillary methods have the potential to provide insight, at the single cell level, into predator–prey relationships in phagotrophic eukaryotes, uncover prey associated phages and intracellular symbionts, and identify viral infections. On the other hand, technical issues associated with separation of single cells and genome amplification cannot be ignored. For example, associations may be wrongly inferred if DNA from non-associated entities is introduced into a sample by co-sorting under relaxed sorting conditions or if bacteria/viruses are firmly attached to cells [38]. Another critical step—whole genome amplification—may introduce various artefacts including amplification bias and genome loss [39]. The most challenging aspect, however, is the specific interpretation of the nature of intracellular associations revealed by SCG. In data obtained from phagocytosis-capable eukaryotes (table 1), it has been difficult to discern whether bacterial contigs in SCG assemblies belonged to prey items or endosymbionts. This situation may not be simpler for photosynthetic microbial eukaryotes, as many of them are also capable of phagocytosis [40]. Distinguishing prey from other intracellular entities *in silico* is of considerable importance or else long-term interactions may remain obscure because of this uncertainty. Even if viruses (only double stranded DNA and single stranded DNA viruses are detectable by SCG) or strictly intracellularly living organisms (e.g. members of Chlamydiae or Rickettsiales) are detected, their origin as prey items cannot be renounced. Clustered regularly interspaced short palindromic repeat (CRISPR) systems can link viruses to their host but this approach has two limitations: it is only suited for bacterial/archaeal genomes, and complete or nearly complete genome assemblies from host and virus are necessary [41,42]. Thus, while using CRISPRs can link viruses with bacteria and archaea, either food or symbiotic, it relies on the quality of the assemblies. Alternatively, putative viral-host connections can be identified by aligning viral transfer RNAs with other recovered genomes [43].

**Table 1.** Unicellular eukaryote single-cell genomic studies revealing intracellular associations.

eukaryote cell sequenced (taxonomic affiliation)	no. of cells sequenced	intracellular entities revealed^a^	reference
Picozoa (Diaphoretickes)	3	Bacteroidetes, Proteobacteria, Firmicutes, nanovirus, large double stranded DNA viruses, Proteobacteria, phages	[26]
*Emiliania huxleyi* (Haptista)	84	*E*. *huxleyi*-virus (EhV)	[29]
MAST-4 lineage (Stramenopiles)	1	*Pelagibacter ubique*	[28]
Chrysophyta (Stramenopiles)	3	Actinobacteria; Bacteroidetes; bacteria related to the candidate divisions TG2 and ZV3	[28]
Basidiomycota (Opisthokonta)	1	Bacteroidetes	[28]
Alveolata	1	Bacteroidetes	[28]
Cercozoa (Rhizaria)	1	bacteria distantly related to Rickettsiales	[28]
*Paulinella* cf. *ovalis* (Cercozoa)	2	Cyanobacteria, cyanophages, Proteobacteria	[36]
*Paramecium aurelia* (Ciliophora)	2	Proteobacteria, Actinobacteria, Cyanobacteria, Firmicutes, Bacteroidetes	[35]

^a^It cannot be ruled out that in some instances the listed intracellular entities may represent accidentally co-sorted bacteria, archaea or viruses.

A complementary approach to differentiate free-living and endosymbiotic organisms is to look for typical genomic signatures of an intracellular lifestyle. Generally speaking, bacteria obligately associated with and living intracellularly in other organisms tend to have genomes that are (i) small, (ii) AT-rich, and (iii) rapidly evolving [44,45]. These genome changes occur during the transitions from free-living to facultative to obligately intracellular [46]. In the early stage of the adaptation towards endosymbiosis, i.e. during the shift from a free-living to an intracellular lifestyle, genomes undergo extensive pseudogenization and gene loss [47,48]. Further adaptation towards obligate endosymbiosis, hence towards a stable and nutrient-rich environment, leads to genome reduction through gene loss. Endosymbionts tend to have limited biosynthetic capabilities (though a unique combination of functional pathways is often retained [46]) and pathways of energy metabolism and biosynthesis of nucleotides, amino acids or vitamins are the most frequently disrupted [44]. Genome reduction affects many other genes, including those involved in cell motility [49], cell division [50], or DNA repair mechanisms [51,52]. In addition, their genes frequently encode a wide range of proteins involved in host interactions or the transport of metabolites [8,53]. Pathogens have acquired genes encoding proteins with eukaryotic motifs that are probably involved in modulation of host functions, e.g. suppressing host defences and allowing survival and replication within eukaryotic host cells [54]. Taken together, a wealth of genomic signatures is associated with the intracellular lifestyle; these signatures provide an opportunity to build and gradually optimize models for predicting endosymbionts in SCG data. Considering that some genomic signatures indicate well-defined effects of symbiont presence on host fitness (e.g. pathogens, metabolic mutualists), or stages of host adaptation, the models may ideally provide even finer classification.

## 4. Validating the nature of intracellular associations revealed by single-cell genomics

While the aforementioned possible strategies for the distinction between prey and other intracellular entities heavily rely on the genomic sequence data, other approaches can be used to further validate the nature of intracellular associations revealed by SCG. Recurrent observation of the same ‘contaminating’ DNA may suggest the symbiotic lifestyle and rule out the prey origin, especially in combination with starvation experiments. However, it is nearly impossible to eliminate bacteria in environmental samples without depletion of heterotrophic unicellular eukaryotes. A plausible strategy could be the replacement of a diverse bacterial community by a homogeneous and well-defined population of a single bacterium. Lagkouvardos *et al*. [55] succeeded using the *Escherichia coli* tolC knockout strain that not only substituted diverse original bacterial communities in several environmental isolates of *Acanthamoeba* but also could be later controlled by a sublethal concentration of ampicillin owing to its hypersensitivity.

Distinction between prey and endosymbionts can also be achieved by localization of the organism(s) detected in the single-cell data using fluorescence *in situ* hybridization via specific oligonucleotide probes. The sample has to be chemically fixed [56] for later hybridization and microscopical inspection. Endosymbionts may reside virtually in any possible compartment within the host cell whereas phagocytized prey is generally limited only to phagosomes and last until completely digested in phagolysosome (phagosome fused with lysosomes). Thus, an intracellular localization beyond vacuoles provides conclusive evidence for the symbiotic lifestyle whereas the presence within vacuoles does not prove the prey origin. Both endosymbionts and prey can be found in phagosomes because phagocytosis is also a common way of entering host cells for endosymbionts [57]. Even the presence within acidified vacuoles (late phagosomes) does not necessarily confirm a prey origin as some pathogens are known for their ability to persist within acidified vacuoles (e.g. *Coxiella burnetii* [58]).

Lastly, single-cell RNA sequencing (scRNA-seq) provides an opportunity to assess the nature of intracellular associations. Though still hampered by technical challenges when applied to single celled eukaryotic microorganisms [59], progress has been made over the last years (e.g. [60–63]). Because scRNA-seq is capable of showing different patterns of gene expression in each cell at a specific time point, it could be highly beneficial in the differentiation of symbionts versus prey. Unlike the adapted endosymbionts able to manipulate phagosome maturation, phagocytized free-living organisms encounter a hostile environment with which they cannot cope. The phagosome becomes more acidic during its maturation and the engulfed microorganisms are gradually degraded by hydrolytic enzymes [64]. Thus, they cannot optimally function which would be reflected in their gene expression. Consequently, apart from the eukaryote host transcriptome probably represented in high abundance in scRNA-seq data, endosymbiont transcripts could be present with upregulated genes involved in host interactions. One caveat to this approach however is that current scRNA-seq protocols applied to single celled eukaryotic microorganisms [60–63] do not process all RNA present in the eukaryotic host cell. Instead, only eukaryotic messenger RNAs (mRNAs) that possess polyadenylated (polyA) tails are hybridized to an oligo(dT)-containing primer and transcribed into complementary DNA [65]. Thus, ribosomal RNAs (rRNAs) and transfer RNAs along with bacterial or archaeal mRNAs are omitted from sequencing. While scRNA-seq has been applied to bacteria [66], there is no rRNA depletion step [67], hampering efficient transcript detection. An alternative approach to the polyA selection universal for both host and symbiont cells is needed.

## 5. Concluding remarks

Intracellular associations underlie the emergence and subsequent flourishing and evolutionary trajectory of eukaryotic cells. However, as pointed out by Woyke & Schulz [68], we still do not know much about these intimate relationships, and what we do know is heavily skewed towards established laboratory systems, represented by a small number of model hosts that do not reflect the true diversity of eukaryotes (figure 1) or their intracellular associations. We predict that SCG methods will lead to fundamental discoveries and move us forward in understanding one of the most remarkable phenomena on Earth.

In principle, there are two primary directions for exploring diverse eukaryotic lineages and their associated intracellular entities. First, microbial culture collections around the world store strains of unicellular eukaryotes, some of which represent neglected eukaryotic lineages [69]. Most likely, reasons for their exclusion as established model taxa include difficulties with maintaining these cultures. Their genome data could be obtained by SCG, and priority strains with genomic signatures of likely symbionts or viruses could be selected to optimize culture conditions for further experimental work. The second approach would encompass the targeted sorting of unicellular eukaryotes from environmental samples with a focus on eukaryotic lineages currently lacking in culture collections.

To accelerate progress in this field, we encourage the research community to establish a genomic encyclopaedia of eukaryote intracellular associations. A robust genomic database connecting eukaryote host cells and their intracellular entities will enable (i) extension of the tree of life with new microbes (archaeal, bacterial, and eukaryotic) and viruses, (ii) investigation of the intracellular associations in ecological context (determination of prevalence, distribution and abundance of identified associations), and (iii) large-scale comparative genome analyses (metabolic features, virulence factors, new gene functions provided to hosts). By sequencing a single eukaryotic cell at a time, we can bypass cultivation requirements, overcome existing biases and gain insight into naturally occurring associations within so far neglected eukaryotic hosts.

## Data accessibility

This article has no additional data.

## References

[1] Martijn J, Vosseberg J, Guy L, Offre P, Ettema TJG 2018 Deep mitochondrial origin outside the sampled alphaproteobacteria. Nature 557, 101–105. (10.1038/s41586-018-0059-5) 29695865

[2] Ponce-Toledo RI, Deschamps P, López-García P, Zivanovic Y, Benzerara K, Moreira D 2017 An early-branching freshwater cyanobacterium at the origin of plastids. Curr. Biol. 27, 386–391. (10.1016/j.cub.2016.11.056) 28132810 PMC5650054

[3] Füssy Z, Oborník M 2018 Complex endosymbioses I: from primary to complex plastids, multiple independent events. In Plastids. Methods in molecular biology (ed. M Maréchal), pp. 17–35. New York, NY: Humana Press. 10.1007/978-1-4939-8654-5_2 29987712

[4] Gilson PR, McFadden GI 2002 Jam packed genomes—a preliminary, comparative analysis of nucleomorphs. Genetica 115, 13–28. (10.1023/a:1016011812442) 12188044

[5] Archibald JM 2007 Nucleomorph genomes: structure, function, origin and evolution. Bioessays 29, 392–402. (10.1002/bies.20551) 17373660

[6] Imanian B, Pombert J-F, Dorrell RG, Burki F, Keeling PJ 2012 Tertiary endosymbiosis in two dinotoms has generated little change in the mitochondrial genomes of their dinoflagellate hosts and diatom endosymbionts. PLoS ONE 7, e43763 (10.1371/journal.pone.0043763) 22916303 PMC3423374

[7] Martin BD, Schwab E 2012 Current usage of symbiosis and associated terminology. Int. J. Biol. 5, 32–45. (10.5539/ijb.v5n1p32)

[8] Tanifuji G et al 2011 Genomic characterization of *Neoparamoeba pemaquidensis* (Amoebozoa) and its kinetoplastid endosymbiont. Eukaryot. Cell 10, 1143–1146. (10.1128/ec.05027-11) 21666073 PMC3165438

[9] Suttle CA 2007 Marine viruses—major players in the global ecosystem. Nat. Rev. Microbiol. 5, 801–812. (10.1038/nrmicro1750) 17853907

[10] Koonin EV, Yutin N 2018 Multiple evolutionary origins of giant viruses. F1000Res. 7, 1840 (10.12688/f1000research.16248.1) PMC6259494 30542614

[11] La Scola B et al 2008 The virophage as a unique parasite of the giant mimivirus. Nature 455, 100–104. (10.1038/nature07218) 18690211

[12] Schulz F et al 2017 Giant viruses with an expanded complement of translation system components. Science 356, 82–85. (10.1126/science.aal4657) 28386012

[13] Rowbotham TJ 1980 Preliminary report on the pathogenicity of *Legionella pneumophila* for freshwater and soil amoebae. J. Clin. Pathol. 33, 1179–1183. (10.1136/jcp.33.12.1179) 7451664 PMC1146371

[14] Tosetti N, Croxatto A, Greub G 2014 Amoebae as a tool to isolate new bacterial species, to discover new virulence factors and to study the host–pathogen interactions. Microb. Pathog. 77, 125–130. (10.1016/j.micpath.2014.07.009) 25088032

[15] Abergel C, Legendre M, Claverie J-M 2015 The rapidly expanding universe of giant viruses: mimivirus, pandoravirus, pithovirus and mollivirus. FEMS Microbiol. Rev. 39, 779–796. (10.1093/femsre/fuv037) 26391910

[16] Taylor-Brown A, Vaughan L, Greub G, Timms P, Polkinghorne A 2014 Twenty years of research into *Chlamydia*-like organisms: a revolution in our understanding of the biology and pathogenicity of members of the phylum Chlamydiae. Pathog. Dis. 73, 1–15. (10.1093/femspd/ftu009) 25854000

[17] Szokoli F, Castelli M, Sabaneyeva E, Schrallhammer M, Krenek S, Doak TG, Berendonk TU, Petroni G 2016 Disentangling the taxonomy of *Rickettsiales* and description of two novel symbionts (‘*Candidatus* Bealeia paramacronuclearis’ and ‘*Candidatus* Fokinia cryptica’) sharing the cytoplasm of the ciliate protist *Paramecium biaurelia*. Appl. Environ. Microbiol. 82, 7236–7247. (10.1128/aem.02284-16) 27742680 PMC5118934

[18] Pillonel T, Bertelli C, Greub G 2018 Environmental metagenomic assemblies reveal seven new highly divergent chlamydial lineages and hallmarks of a conserved intracellular lifestyle. Front. Microbiol. 9, 79 (10.3389/fmicb.2018.00079) 29515524 PMC5826181

[19] Schulz F, Alteio L, Goudeau D, Ryan EM, Yu FB, Malmstrom RR, Blanchard J, Woyke T 2018 Hidden diversity of soil giant viruses. Nat. Commun. 9, 4881 (10.1038/s41467-018-07335-2) 30451857 PMC6243002

[20] Nowack ECM, Melkonian M 2010 Endosymbiotic associations within protists. Phil. Trans. R. Soc. B 365, 699–712. (10.1098/rstb.2009.0188) 20124339 PMC2817226

[21] Adl SM et al 2018 Revisions to the classification, nomenclature, and diversity of eukaryotes. J. Eukaryot. Microbiol. 66, 4–119. (10.1111/jeu.12691) PMC6492006 30257078

[22] Stepanauskas R 2012 Single cell genomics: an individual look at microbes. Curr. Opin. Microbiol. 15, 613–620. (10.1016/j.mib.2012.09.001) 23026140

[23] Woyke T, Doud DFR, Schulz F 2017 The trajectory of microbial single-cell sequencing. Nat. Methods 14, 1045–1054. (10.1038/nmeth.4469) 29088131

[24] Rinke C et al 2013 Insights into the phylogeny and coding potential of microbial dark matter. Nature 499, 431–437. (10.1038/nature12352) 23851394

[25] Marcy Y et al 2007 Dissecting biological ‘dark matter’ with single-cell genetic analysis of rare and uncultivated TM7 microbes from the human mouth. Proc. Natl Acad. Sci. USA 104, 11 889–11 894. (10.1073/pnas.0704662104) PMC1924555 17620602

[26] Yoon HS, Price DC, Stepanauskas R, Rajah VD, Sieracki ME, Wilson WH, Yang EC, Duffy S, Bhattacharya D 2011 Single-cell genomics reveals organismal interactions in uncultivated marine protists. Science 332, 714–717. (10.1126/science.1203163) 21551060

[27] Caron DA, Countway PD, Jones AC, Kim DY, Schnetzer A 2012 Marine protistan diversity. Annu. Rev. Mar. Sci. 4, 467–493. (10.1146/annurev-marine-120709-142802) 22457984

[28] Martinez-Garcia M, Brazel D, Poulton NJ, Swan BK, Gomez ML, Masland D, Sieracki ME, Stepanauskas R 2012 Unveiling *in situ* interactions between marine protists and bacteria through single cell sequencing. ISME J. 6, 703–707. (10.1038/ismej.2011.126) 21938022 PMC3280149

[29] Martínez-Martínez J, Poulton NJ, Stepanauskas R, Sieracki ME, Wilson WH 2011 Targeted sorting of single virus-infected cells of the coccolithophore *Emiliania huxleyi*. PLoS ONE 6, e22520 (10.1371/journal.pone.0022520) 21818332 PMC3144233

[30] Roy RS, Price DC, Schliep A, Cai G, Korobeynikov A, Yoon HS, Yang EC, Bhattacharya D 2014 Single cell genome analysis of an uncultured heterotrophic stramenopile. Sci. Rep. 4, 4780 (10.1038/srep04780) 24759094 PMC3998028

[31] Gawryluk RMR, del Campo J, Okamoto N, Strassert JFH, Lukeš J, Richards TA, Worden AZ, Santoro AE, Keeling PJ. 2016 Morphological identification and single-cell genomics of marine diplonemids. Curr. Biol. 26, 3053–3059. (10.1016/j.cub.2016.09.013) 27875688

[32] Seeleuthner Y et al 2018 Single-cell genomics of multiple uncultured stramenopiles reveals underestimated functional diversity across oceans. Nat. Commun. 9, 310 (10.1038/s41467-017-02235-3) 29358710 PMC5778133

[33] Blainey PC 2013 The future is now: single-cell genomics of bacteria and archaea. FEMS Microbiol. Rev. 37, 407–427. (10.1111/1574-6976.12015) 23298390 PMC3878092

[34] Hongoh Y et al 2008 Complete genome of the uncultured Termite Group 1 bacteria in a single host protist cell. Proc. Natl Acad. Sci. USA 105, 5555–5560. (10.1073/pnas.0801389105) 18391199 PMC2291132

[35] Not F, Gausling R, Azam F, Heidelberg JF, Worden AZ 2007 Vertical distribution of picoeukaryotic diversity in the Sargasso Sea. Environ. Microbiol. 9, 1233–1252. (10.1111/j.1462-2920.2007.01247.x) 17472637

[36] Bhattacharya D, Price DC, Yoon HS, Yang EC, Poulton NJ, Andersen RA, Das SP 2012 Single cell genome analysis supports a link between phagotrophy and primary plastid endosymbiosis. Sci. Rep. 2, 356 (10.1038/srep00356) 22493757 PMC3322482

[37] Plotnikov AO, Balkin AS, Gogoleva NE, Lanzoni O, Khlopko YA, Cherkasov SV, Potekhin AA 2019 High-throughput sequencing of the 16S rRNA gene as a survey to analyze the microbiomes of free-living ciliates *Paramecium*. Microb. Ecol. 78, 286–298. (10.1007/s00248-019-01321-x) 30661111

[38] Worden AZ, Dupont C, Allen AE 2011 Genomes of uncultured eukaryotes: sorting FACS from fiction. Genome Biol. 12, 117 (10.1186/gb-2011-12-6-117) 21722350 PMC3218834

[39] Gawad C, Koh W, Quake SR 2016 Single-cell genome sequencing: current state of the science. Nat. Rev. Genet. 17, 175–188. (10.1038/nrg.2015.16) 26806412

[40] Hartmann M, Grob C, Tarran GA, Martin AP, Burkill PH, Scanlan DJ, Zubkov MV 2012 Mixotrophic basis of Atlantic oligotrophic ecosystems. Proc. Natl Acad. Sci. USA 109, 5756–5760. (10.1073/pnas.1118179109) 22451938 PMC3326507

[41] Andersson AF, Banfield JF 2008 Virus population dynamics and acquired virus resistance in natural microbial communities. Science 320, 1047–1050. (10.1126/science.1157358) 18497291

[42] Anderson RE, Brazelton WJ, Baross JA 2011 Using CRISPRs as a metagenomic tool to identify microbial hosts of a diffuse flow hydrothermal vent viral assemblage. FEMS Microbiol. Ecol. 77, 120–133. (10.1111/j.1574-6941.2011.01090.x) 21410492

[43] Paez-Espino D, Eloe-Fadrosh EA, Pavlopoulos GA, Thomas AD, Huntemann M, Mikhailova N, Rubin E, Ivanova NN, Kyrpides NC 2016 Uncovering Earth's virome. Nature 536, 425–430. (10.1038/nature19094) 27533034

[44] Moran NA, McCutcheon JP, Nakabachi A 2008 Genomics and evolution of heritable bacterial symbionts. Annu. Rev. Genet. 42, 165–190. (10.1146/annurev.genet.41.110306.130119) 18983256

[45] Land M et al 2015 Insights from 20 years of bacterial genome sequencing. Funct. Integr. Genomics 15, 141–161. (10.1007/s10142-015-0433-4) 25722247 PMC4361730

[46] Moran NA 2002 Microbial minimalism. Cell 108, 583–586. (10.1016/s0092-8674(02)00665-7) 11893328

[47] Toft C, Andersson SGE 2010 Evolutionary microbial genomics: insights into bacterial host adaptation. Nat. Rev. Genet. 11, 465–475. (10.1038/nrg2798) 20517341

[48] Lind AE, Lewis WH, Spang A, Guy L, Embley TM, Ettema TJG 2018 Genomes of two archaeal endosymbionts show convergent adaptations to an intracellular lifestyle. ISME J. 12, 2655–2667. (10.1038/s41396-018-0207-9) 29991760 PMC6194110

[49] Toft C, Fares MA 2008 The evolution of the flagellar assembly pathway in endosymbiotic bacterial genomes. Mol. Biol. Evol. 25, 2069–2076. (10.1093/molbev/msn153) 18635679

[50] Erickson HP, Osawa M 2010 Cell division without FtsZ—a variety of redundant mechanisms. Mol. Microbiol. 78, 267–270. (10.1111/j.1365-2958.2010.07321.x) 20979330 PMC2966023

[51] Gil R et al 2003 The genome sequence of *Blochmannia floridanus*: comparative analysis of reduced genomes. Proc. Natl Acad. Sci. USA 100, 9388–9393. (10.1073/pnas.1533499100) 12886019 PMC170928

[52] Nowack ECM, Melkonian M, Glöckner G 2008 Chromatophore genome sequence of *Paulinella* sheds light on acquisition of photosynthesis by eukaryotes. Curr. Biol. 18, 410–418. (10.1016/j.cub.2008.02.051) 18356055

[53] Schmitz-Esser S, Tischler P, Arnold R, Montanaro J, Wagner M, Rattei T, Horn M 2009 The genome of the amoeba symbiont ‘*Candidatus* Amoebophilus asiaticus’ reveals common mechanisms for host cell interaction among amoeba-associated bacteria. J. Bacteriol. 192, 1045–1057. (10.1128/jb.01379-09) 20023027 PMC2812958

[54] Gomez-Valero L et al 2019 More than 18,000 effectors in the *Legionella* genus genome provide multiple, independent combinations for replication in human cells. Proc. Natl Acad. Sci. USA 116, 2265–2273. (10.1073/pnas.1808016116) 30659146 PMC6369783

[55] Lagkouvardos I, Shen J, Horn M 2014 Improved axenization method reveals complexity of symbiotic associations between bacteria and acanthamoebae. Environ. Microbiol. Rep. 6, 383–388. (10.1111/1758-2229.12162) 24992537

[56] Amann R, Fuchs BM 2008 Single-cell identification in microbial communities by improved fluorescence *in situ* hybridization techniques. Nat. Rev. Microbiol. 6, 339–348. (10.1038/nrmicro1888) 18414500

[57] Casadevall A 2008 Evolution of intracellular pathogens. Annu. Rev. Microbiol. 62, 19–33. (10.1146/annurev.micro.61.080706.093305) 18785836

[58] Maurin M, Benoliel AM, Bongrand P, Raoult D 1992 Phagolysosomes of *Coxiella burnetii*-infected cell lines maintain an acidic pH during persistent infection. Infect. Immun. 60, 5013–5016. 1452331 10.1128/iai.60.12.5013-5016.1992 PMC258270

[59] Liu Z, Hu SK, Campbell V, Tatters AO, Heidelberg KB, Caron DA 2017 Single-cell transcriptomics of small microbial eukaryotes: limitations and potential. ISME J. 11, 1282–1285. (10.1038/ismej.2016.190) 28060364 PMC5437924

[60] Kolisko M, Boscaro V, Burki F, Lynn DH, Keeling PJ 2014 Single-cell transcriptomics for microbial eukaryotes. Curr. Biol. 24, R1081–R1082. (10.1016/j.cub.2014.10.026) 25458215

[61] Krabberød AK, Orr RJS, Bråte J, Kristensen T, Bjørklund KR, Shalchian-Tabrizi K 2017 Single cell transcriptomics, mega-phylogeny, and the genetic basis of morphological innovations in Rhizaria. Mol. Biol. Evol. 34, 1557–1573. (10.1093/molbev/msx075) 28333264 PMC5455982

[62] Lax G, Eglit Y, Eme L, Bertrand EM, Roger AJ, Simpson AGB 2018 Hemimastigophora is a novel supra-kingdom-level lineage of eukaryotes. Nature 564, 410–414. (10.1038/s41586-018-0708-8) 30429611

[63] Vacek V, Novák LVF, Treitli SC, Táborský P, Čepička I, Kolísko M, Keeling PJ, Hampl V 2018 Fe–S cluster assembly in oxymonads and related protists. Mol. Biol. Evol. 35, 2712–2718. (10.1093/molbev/msy168) 30184127 PMC6231488

[64] Smith LM, May RC 2013 Mechanisms of microbial escape from phagocyte killing. Biochem. Soc. Trans. 41, 475–490. (10.1042/bst20130014) 23514140

[65] Picelli S, Björklund ÅK, Faridani OR, Sagasser S, Winberg G, Sandberg R 2013 Smart-seq2 for sensitive full-length transcriptome profiling in single cells. Nat. Methods 10, 1096–1098. (10.1038/nmeth.2639) 24056875

[66] Kang Y, McMillan I, Norris MH, Hoang TT 2015 Single prokaryotic cell isolation and total transcript amplification protocol for transcriptomic analysis. Nat. Protoc. 10, 974–984. (10.1038/nprot.2015.058) 26042386 PMC4494743

[67] Zhao S, Zhang Y, Gamini R, Zhang B, von Schack D. 2018 Evaluation of two main RNA-seq approaches for gene quantification in clinical RNA sequencing: polyA+ selection versus rRNA depletion. Sci. Rep. 8, 4781 (10.1038/s41598-018-23226-4) 29556074 PMC5859127

[68] Woyke T, Schulz F 2018 Entities inside one another—a matryoshka doll in biology? Environ. Microbiol. Rep. 11, 26–28. (10.1111/1758-2229.12716) 30588764 PMC7379638

[69] del Campo J, Sieracki ME, Molestina R, Keeling P, Massana R, Ruiz-Trillo I 2014 The others: our biased perspective of eukaryotic genomes. Trends Ecol. Evol. 29, 252–259. (10.1016/j.tree.2014.03.006) 24726347 PMC4342545

